# Why I joined the research laboratory of Prof. D. Carleton Gajdusek in 1968

**DOI:** 10.1098/rstb.2008.4008

**Published:** 2008-11-27

**Authors:** Françoise Cathala

**Affiliations:** 68 Boulevard St Michel75006 Paris, France

Two important milestones in my life: I was born in 1921 to a military family from Languedoc in France; in 1986 I took compulsory retirement after declining to create a new research unit in the Pitié Salpétrière Hospital in Paris. Sometime in between I discovered what hospital life was like, following the disaster of 1940!

Because I was for 1 year an auxiliary medical officer in the First Army, in 1945 I had the opportunity to apply for a position in the Hôpitaux de Paris, and after joining this organization I remained there until my retirement. I was in the Institut National de la Recherche Scientifique from its inception. At the beginning I made the choice to work in neurology and virology. I was trained at the Institut Pasteur under the prestigious supervision of André Lwoff. In my work, I have always had the strong support of Prof. Paul Castaigne. As well as being a doctor in the Hôpitaux de Paris and a graduate of the Institut Pasteur, I am a member of the French Society of Neurology. However, I did not initially embark on an independent career because my husband was building up his own.

Why the research laboratory of Prof. D. Carleton Gajdusek? To this question from a researcher at the National Institutes of Health in Bethesda, Maryland I answered, ‘Owing to his publications’. I was fascinated by the notion of a latent virus in the nervous system after a primary infection, with occasional manifestations appearing subsequently, a phenomenon well known with herpes simplex virus and the herpes virus of varicella–zoster, which had been the subject of my thesis. I wanted to apply research on slow virus diseases eventually to study multiple sclerosis, without having then any knowledge at all about kuru or the transmission of Creutzfeldt–Jakob disease to chimpanzees!

My first experience of the role of a virus in neurology was a cruel failure but ultimately had some benefits for our research programme. I was working as an assistant in a neurosurgical unit in the morning and in a microbiology laboratory in the afternoon. I saw a young patient with a significantly high level of antibodies against measles in the cerebrospinal fluid (CSF). So I asked for and obtained a cerebral biopsy with the intention of setting up cell cultures and inoculating animals. However, this opportunistic experimental study displeased my superior, who threw away my biopsy. The following day, as I was shouting with rage, a young neuropathologist, Michel Bouteille, heard me and, after the necropsy of the patient, examined the brain under electron microscopy. He found measles virus, as had been described by Gabriele Zu Rhein. It was a case of subacute sclerosing panencephalitis.

While working in the same unit, I found another patient of interest: a case of transverse myelitis, with a raised level of antibodies against measles in the CSF. From this patient I had the idea that testing the CSF for anti-measles antibodies in patients with multiple sclerosis would be an interesting line to pursue. In the service of Paul Castaigne, my colleague Edmond Schuller had become the specialist in the study of proteins in the CSF and we took many samples from our patients. It was with this CSF collection that I left to work in the laboratory of Carleton Gajdusek—and in due course this led to my first publication with Paul Brown.

Well accepted by the members of Carleton's laboratory, I took part in their activities and, at the same time, I was informed about the investigations on kuru and Creutzfeldt–Jakob disease, which had then just been passed to the chimpanzee. The guided tour by Joe Gibbs of the animal house at Patuxent was for me a revelation! I was stunned to see these animals at different stages of disease, either of kuru or Creutzfeldt–Jakob disease. I could not avoid making comparison with the regular clinical rounds of the consulting team at the Pitié Salpétrière Hospital, where poor patients with neurological diseases were admitted. Then, as I saw more, I became acutely aware of the importance of these transmission studies in chimpanzees.

A second surprise I had was seeing a movie on scrapie in sheep. Of course I had seen this disease in the flock on our farm in Languedoc. When I returned for Christmas holidays in Languedoc, the owner of the sheep flock assured me that scrapie was common and that everybody consumed such sheep without risk. It was impossible not to see the connection between the movie and the presence of scrapie in France.

It was during the same year that my father-in-law, Prof. Jean Cathala, died. After the death of my father at the time of the Liberation in France, I had transferred my affection to my father-in-law, in admiration both of his professional standing and his phenomenal breadth of knowledge. When he realized this, Dr Gajdusek invited me to spend the last two months of my stay at his home so that I would not have to live alone.

My last delightful discovery was Carleton's house filled with objects from the South Pacific and all these boys from Papua New Guinea and Micronesia educated and kept on a tight rein by Joe Wegstein, who shared the house with Carleton. It was really another way of life by comparison with my own family brought up in the Protestant tradition of the Cévennes. However, in other ways I was not so surprised because we too have known the separation of children from their families for the purposes of education. Furthermore, the giving of thanks before the meal in the evening reminded me of our own practices. I became attached to Carleton's family and came there again frequently; some of the children have also come to visit me in my home.

After I had returned to France, I managed to organize my life so that I could work in the small laboratory of Prof. Castaigne on the isolation of viruses in cell cultures and, in the other half of my time, perform my first experiments inoculating primates, supervised by Carleton Gajdusek and Joe Gibbs. I cannot describe here all my work but in our *culturepasteurienne* the first aim always was to verify Koch's postulates; therefore to demonstrate a new pathogen as the cause of a disease, it was necessary to reproduce this disease by the same experiments carried out elsewhere by independent investigators. This was done, and these studies were considered important in France. Moreover, it was through this means that my husband introduced me to his colleague and friend, the military doctor Louis Court.

Nevertheless, my career did not improve! To get ahead it was necessary to become Maître de Recherches. For this reason I moved to Lyon. I will not speak about these years in Lyon except that it was during this time, in 1976, that Carleton Gajdusek was awarded his Nobel Prize.

I took advantage of this opportunity to return to Paris, by collaborating with a colleague from the Institut Pasteur. Conscious that I would not be able to find the means of supporting an efficient laboratory, I suggested to Carleton that I should do an epidemiological study of Creutzfeldt–Jakob disease because France was without doubt ideal for this. Carleton sent me Paul Brown to carry out this study. Paul proceeded to marry my niece and became my nephew—and our epidemiological research prospered. The success of an experimental study at the military hospital of Percy–Clamart with transmission to the chimpanzee of a French strain of Creutzfeldt–Jakob disease from Strasbourg led Louis Court and his team to be deeply involved in this research, which became his main concern, coupled with the organization of many successful international symposiums. He was strongly supported in this work by the Centre de Recherche de Grenoble and the Institut Mérieux de Lyon.

During the same period, my colleague at the Institut Pasteur became interested in scrapie in sheep. She also enabled our American colleagues to report the first transmission of familial Creutzfeldt–Jakob disease. Despite this evidence, the research establishment in France remained unconvinced. However, they were obliged to take note of the importance of this programme when, in 1996, the English scientists attending a symposium organized by Louis Court at the Military Hospital of Val de Grâce in Paris were called back urgently to the UK. The reason was the announcement of the risk of transmission to humans of a new bovine disease (bovine spongiform encephalopathy) perhaps due to the scrapie agent!

Time had also gone by. Weary of so much effort and the sacrifices imposed on my family, I declined the proposal from Jean-Pierre Changeux to create my own research unit. I considered that it was too late to start this at my age. Moreover, Dominique Dormont, who was then in charge of the Laboratory of the Military Hospital, was competent and perfectly capable of carrying out this research.

Thunderstorms were forecast on the horizon. I followed Paul Castaigne's wise advice: ‘Come back home.’

But after that, what then? Why did I join Gajdusek in the first place? It was owing to the possibility of a viral origin of multiple sclerosis. Why did I remain closely associated with him? It was owing to his exceptional programme of research that I am proud to have had a part in. Finally, and most importantly, it was owing to the friendships that I found there, which I continue to hold most dear.

